# Metabolic engineering of *Vibrio natriegens* for anaerobic succinate production

**DOI:** 10.1111/1751-7915.13983

**Published:** 2021-11-29

**Authors:** Felix Thoma, Clarissa Schulze, Carolina Gutierrez‐Coto, Maurice Hädrich, Janine Huber, Christoph Gunkel, Rebecca Thoma, Bastian Blombach

**Affiliations:** ^1^ Microbial Biotechnology Campus Straubing for Biotechnology and Sustainability Technical University of Munich Uferstraße 53 Straubing 94315 Germany; ^2^ SynBiofoundry@TUM Campus Straubing for Biotechnology and Sustainability Technical University of Munich Schulgasse 22 Straubing 94315 Germany

## Abstract

The biotechnological production of succinate bears serious potential to fully replace existing petrochemical approaches in the future. In order to establish an economically viable bioprocess, obtaining high titre, yield and productivity is of central importance. In this study, we present a straightforward engineering approach for anaerobic succinate production with *Vibrio natriegens*, consisting of essential metabolic engineering and optimization of process conditions. The final producer strain *V. natriegens* Δ*lldh* Δ*dldh* Δ*pfl* Δ*ald* Δ*dns*::*pyc*_Cg_ (Succ1) yielded 1.46 mol of succinate per mol of glucose under anaerobic conditions (85% of the theoretical maximum) and revealed a particularly high biomass‐specific succinate production rate of 1.33 g_Succ_ g_CDW_^−1^ h^−1^ compared with well‐established production systems. By applying carbon and redox balancing, we determined the intracellular flux distribution and show that under the tested conditions the reductive TCA as well as the oxidative TCA/glyoxylate pathway contributed to succinate formation. In a zero‐growth bioprocess using minimal medium devoid of complex additives and expensive supplements, we obtained a final titre of 60.4 g_Succ_ l^−1^ with a maximum productivity of 20.8 g_Succ_ l^−1^ h^−1^ and an overall volumetric productivity of 8.6 g_Succ_ l^−1^ h^−1^ during the 7 h fermentation. The key performance indicators (titre, yield and productivity) of this first engineering approach in *V. natriegens* are encouraging and compete with costly tailored microbial production systems.

## Graphical abstract

Here we describe the first approach to tailor *Vibrio natriegens* for the anaerobic succinate production from glucose. By the optimization of production conditions and essential metabolic engineering we increased the succinate yield 10‐fold. Finally, we achieved a maximum productivity of 20.8 g l^−1^ h^−1^ in an anaerobic bioprocess with resting cells, highlighting the potential of *V. natriegens* for succinate production.

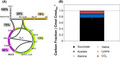

## Introduction

Succinate (1,4‐butanedioate) is a member of the 1,4‐dicarboxylates and was rated amongst the top twelve bio‐based platform chemicals (Werpy and Petersen, 2004; Bozell and Petersen, 2010) since it serves as a precursor for the synthesis of numerous chemical products, e.g. adipate, 1,4‐butanediol, tetrahydrofuran, N‐methylpyrrolidone, 2‐pyrrolidone and γ‐butyrolactone (Lee *et al*., 2019b, 2019c,2019b, 2019c). Its demand is, hitherto, primarily satisfied by the chemical synthesis from the petro‐based precursors butane and benzene, which has been in place for decades (Lee *et al*., 2019b). Motivated by environmental concerns and anticipating a shortage of fossil resources, various studies have demonstrated that the biotechnological production from biogenic resources is feasible and some processes are currently commercialized (Bozell and Petersen, 2010; Ferone *et al*., 2019; Lee *et al*., 2019c). Bioprocesses must achieve superior key performance indicators and produce succinate from inexpensive substrates in cheap media to compete with the petrochemistry. Besides high yield and titre that keep the operating costs at a possible minimum, volumetric productivities can significantly reduce the required fermentation time or scale and thus minimize operating and capital expenditures respectively.

Encouraging data were reported for the bio‐based succinate production with established bacterial (e.g. *Escherichia coli* and *Corynebacterium glutamicum*) and yeast cell systems (e.g. *Saccharomyces cerevisiae* and *Yarrowia lipolytica*), as well as bacteria that naturally secrete large quantities of succinate, such as *Actinobacillus succinogenes*, *Anaerobiospirillum succiniciproducens*, *Mannheimia succiniciproducens* and *Basfia succiniciproducens*. Excellent reviews summarize the wealth of knowledge about metabolic engineering and fermentation strategies of aerobic and anaerobic succinate production (Wieschalka *et al*., 2013; Ahn *et al*., 2016; Ferone *et al*., 2019; Lee *et al*., 2019b; Yang *et al*., 2020; Dai *et al*., 2020; Kumar *et al*., 2020). In general, depending on the host’s metabolism and the anticipated production mode, succinate yields were optimized by the inactivation of competing pathways and the elimination of negative regulation (Lin *et al*., 2005), enhancing e.g. the anaplerotic flux (Vemuri *et al*., 2002a; Thakker *et al*., 2012), NADH availability (Kim *et al*., 2017), the succinate export capacity (Zhu *et al*., 2014), as well as by rational enzyme engineering within the reductive branch of the TCA cycle (Ahn *et al*., 2020). These efforts resulted in engineered strains, that converted glucose (as the sole organic carbon and energy source) efficiently to succinate (≥ 1.40 mol of succinate per mol of glucose (mol_Succ_ mol_Glc_^−1^), ≥ 0.90 g_Succ_ g_Glc_^−1^), and reached final titres of up to 1.24 M (146 g l^−1^) in anaerobic batch and fed‐batch processes (Lee *et al*., 2006; Jantama *et al*., 2008; Okino *et al*., 2008; Ahn *et al*., 2016; Lee *et al*., 2019b; Yang *et al*., 2020; Dai *et al*., 2020). However, only few of these studies reported productivities exceeding 22 mmol succinate l^−1^ h^−1^ (2.5 g l^−1^ h^−1^), which was considered the minimum volumetric productivity to meet economic viability (Werpy and Petersen, 2004).

**Fig. 1:**
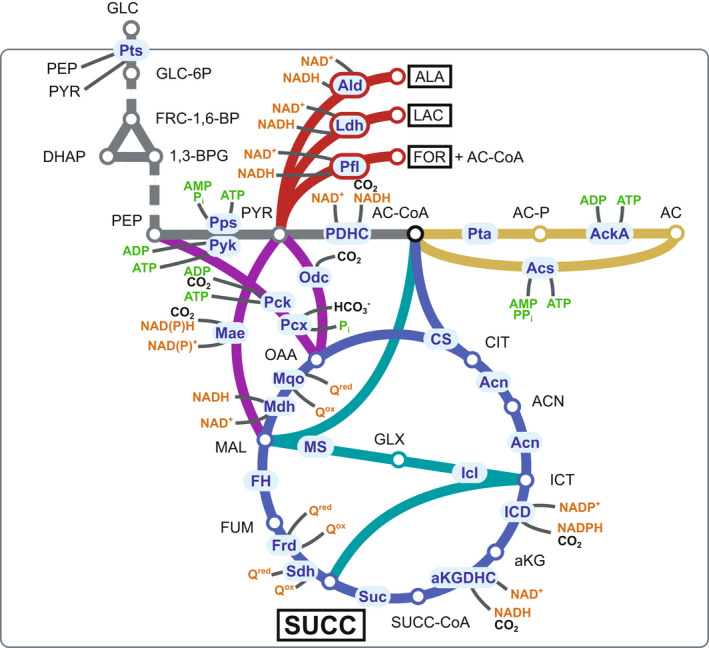
Central metabolic pathways of *V. natriegens*. Enzymatic reactions (shown in blue boxes) are indicated as annotated in the KEGG database. Redox and energetic co‐factors of the reactions are highlighted in orange and green respectively. Prominent products of mixed acid fermentation are boxed. Colour coding: glycolysis, grey; acetate pathway, ochre; pyruvate‐derived fermentation products, red; TCA cycle, blue; glyoxylate pathway, turquoise and anaplerotic reactions, purple. Multiple enzymatic steps are indicated by a dashed line. Inactivated enzymes are framed in red. Abbreviations for metabolites: AC – acetate, AC‐CoA – acetyl‐coenzyme A, ACN – aconitate, AC‐P – acetylphosphate, aKG – α‐ketoglutarate, ALA – alanine, 1,3‐BPG – 1,3‐bisphosphoglycerate, CIT – citrate, DHAP – dihydroxyacetone phosphate, FOR – formate, FRC‐1,6‐BP – fructose‐1,6‐bisphosphate, FUM – fumarate, GLC – glucose, GLC‐6P ‐ glucose‐6‐phosphate, GLX – glyoxylate, ICT – isocitrate, LAC – lactate, MAL – malate, OAA – oxaloacetate, PEP – phosphoenolpyruvate, PYR – pyruvate, Q^ox/red^ – quinone/quinol, SUCC – succinate and SUCC‐CoA – succinyl‐coenzyme A; Abbreviations for enzymes: AckA – acetate kinase, Acn – aconitase, Acs – Acetyl‐CoA synthetase, aKGDHC – α‐ketoglutarate dehydrogenase complex, CS – citrate synthase, FH – fumarate hydratase, Frd – fumarate reductase, ICD – isocitrate dehydrogenase, Icl – isocitrate lyase, Mae – malic enzyme, Mdh – malate dehydrogenase, Mqo – malate:quinone oxidoreductase, MS – malate synthase, Odc – oxaloacetate decarboxylase, Pck – PEP carboxykinase, Pcx – PEP carboxylase, PDHC – pyruvate dehydrogenase complex, Pps – PEP synthase, Pta – phosphate acetyltransferase, Pts – phosphotransferase system, Pyk – pyruvate kinase, Sdh – succinate dehydrogenase and Suc – succinyl‐CoA synthetase.

*Vibrio natriegens* is an attractive candidate for biotechnological succinate production. Its fast growth and high substrate uptake rates (Hoffart *et al*., 2017; Hoff *et al*., 2020; Thoma and Blombach, 2021) set an excellent basis to develop highly productive fermentation processes especially for the bio‐based manufacturing of bulk chemicals such as succinate. The genomic sequence of *V. natriegens* is available and fully annotated (Maida *et al*., 2013; Wang *et al*., 2013; Lee *et al*., 2016). A wealth of molecular biological tools has been established (Lee *et al*., 2016, 2017; Weinstock *et al*., 2016; Dalia *et al*., 2017; Hoffart *et al*., 2017; Tschirhart *et al*., 2019), and flux distributions of central carbon metabolism under aerobic conditions were determined during growth on glucose (Eagon and Wang, 1962; Long *et al*., 2017). This Gram‐negative γ‐proteobacterium is moderately halophilic and grows prototrophically in minimal medium containing glucose under aerobic (µ = 1.48–1.70 h^−1^) and anaerobic conditions (µ = 0.92 h^−1^), and even anaerobically resting cells show a high biomass‐specific glucose consumption rate of 1 g g^−1^ h^−1^ (Thoma and Blombach, 2021). During anaerobic growth, *V. natriegens* ferments glucose to acetate, formate, ethanol, succinate and lactate. Non‐growing anaerobic cells additionally secrete a remarkable amount of alanine (Fig. 1) (Hoffart *et al*., 2017). Deduced from the genome annotation (Lee *et al*., 2016), *V. natriegens* possesses the enzymatic equipment for aerobic and anaerobic succinate production: glycolysis, reductive branch of the TCA cycle (redTCA), oxidative branch of the TCA cycle (oxTCA) and glyoxylate pathway (Fig. 1). Noteworthy, the anaplerotic flux towards oxaloacetate might be exclusively provided via PEP carboxylation in *V. natriegens*. The genome annotation features PEP carboxylase and PEP carboxykinase genes, respectively, whereas a pyruvate carboxylase gene is lacking (Fig. 1). The biotechnological potential of *V. natriegens* has been exemplified recently by demonstrating high productivities of pyruvate‐derived alanine and 2,3‐butanediol, as well as 1,3‐propanediol (Hoffart *et al*., 2017; Erian *et al*., 2020; Zhang *et al*., 2021). However, this bacterium has not been engineered for the production of succinate so far. In this study, we tailored *V. natriegens* for anaerobic succinate production by inactivation of competing pathways, enhancing the anaplerotic flux and optimizing process conditions. In an anaerobic resting cell approach, we produced 60.4 g_Succ_ l^−1^ within 7 h of fermentation, achieving a maximum productivity of 20.8 g_Succ_ l^−1^ h^−1^. The promising results of this first engineering approach highlight the potential of *V. natriegens* as future host for succinate production.

## Results

### Optimum flux distribution for succinate production

**Fig. 2:**
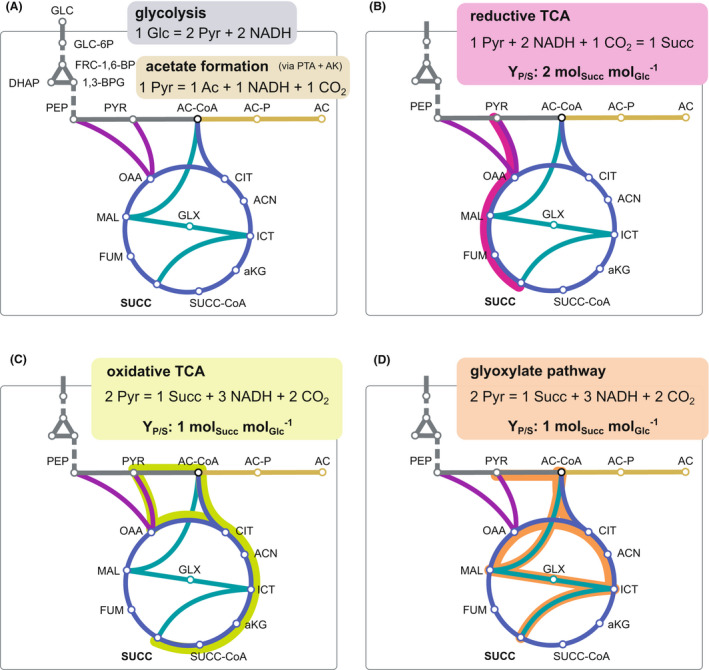
Relevant pathways and overall reaction equations of the different modules (regarding substrate, product, NADH equivalents and CO_2_) for succinate production from glucose in *V. natriegens*. (A) Glucose dissimilation to pyruvate and possible NADH regeneration through acetate formation. Required reactions for succinate production from pyruvate following (B) the reductive branch, (C) the oxidative branch of the TCA cycle and (D) the glyoxylate pathway are highlighted in magenta, green and orange respectively. Abbreviations as in Fig. 1.

Initially, we set up a metabolic network consisting of three equations to describe succinate and acetate production from glucose in *V. natriegens* (Fig. 2). When glucose is solely routed through the glycolysis and redTCA to produce succinate, the maximum product yield (Y_P/S_) of 2 mol_Succ_ mol_Glc_^−1^ can be achieved on the carbon basis (Fig. 2B). However, this requires additional reducing power, as only 2 mol_NADH_ mol_Glc_^−1^ is supplied in glycolysis (Fig. 2A and B). 1 mol_Succ_ mol_Glc_^−1^ is yielded, if succinate is produced via the oxTCA or glyoxylate pathway, but a net surplus of NADH is generated (Fig 2C and D). Since NADH cannot be recycled by the respiratory chain under anaerobic conditions and alternative electron acceptors are not present, the redox state of the cell must be balanced. In the case that oxTCA and glyoxylate pathway are not active under anaerobic conditions, 1.33 mol_Succ_ mol_Glc_^−1^ can be obtained, accompanied by acetate formation with a yield of 0.66 mol_Ac_ mol_Glc_^−1^ (0.50 mol_Ac_ mol_Succ_^−1^). In this scenario, 1 mol pyruvate is decarboxylated by the PDHC to acetyl‐CoA which is subsequently converted to 1 mol acetate yielding 1 mol ATP and NADH respectively. Based on the assumption that all pathways are fully operative under anaerobic conditions, the maximum yield of 1.71 mol_Succ_ mol_Glc_^−1^ (Vuoristo *et al*., 2016) can be achieved by routing 71% of the carbon via the redTCA and 29% via either the oxTCA or the glyoxylate pathway. The oxTCA and glyoxylate pathway perform equally with regard to succinate formation, reduction equivalents and net CO_2_ formation (Fig. 2C and D). Thus, they can functionally compensate each other, and we did not distinguish between the pathways in the following.

### Optimization of production conditions

**Fig. 3:**
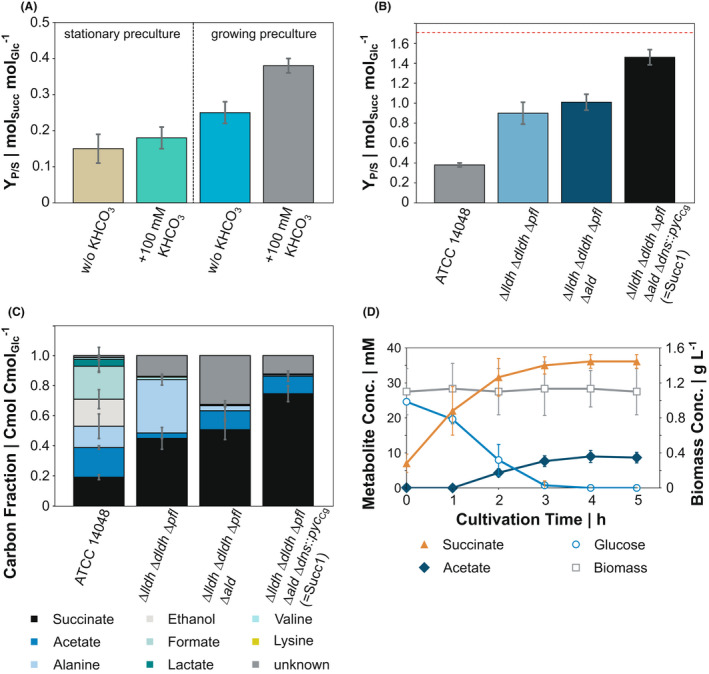
Anaerobic succinate production in test tubes containing 50 ml VN minimal medium with 27.5 mM glucose. Succinate yields on glucose affected (A) by supplementation of 100 mM KHCO_3_ to cultures that were inoculated from stationary phase and growing precultures, respectively, and (B) by metabolic engineering of *V. natriegens*. The red dashed line indicates the theoretical maximum succinate yield on glucose (1.71 mol_Succ_ mol_Glc_^−1^). (C) Carbon balance to access the anaerobic product spectrum and unaccounted by‐products of engineered strains from (B). To avoid an overestimation of the carbon proportion, we calculated only three glucose‐derived carbon atoms for succinate. (D) Succinate production kinetics of *V. natriegens* Succ1 (∆*lldh* ∆*dldh* ∆*pfl* ∆*ald* ∆*dns*::*pyc*_Cg_). All data represent mean values with error bars indicating the standard deviation from at least three independent biological replicates. (C) Standard deviations were calculated by error propagation.

Succinate secretion has been observed as a minor product of fermentative glucose metabolism by anaerobically growing and resting cells of *V. natriegens* (Hoffart *et al*., 2017). In a small anaerobic screening system (50 ml test tubes), we obtained a yield of 0.15 ± 0.04 mol_Succ_ mol_Glc_^−1^ when we cultivated resting cells of *V. natriegens* WT (Fig. 3A). By inoculating the test system from growing precultures, instead of using cells from the stationary phase of overnight (O/N) precultures, we increased the yield by > 60% (Fig. 3A). To provide sufficient HCO_3_^‐^ for the carboxylation of PEP, we supplemented the VN medium additionally with 100 mM KHCO_3_, which further improved the Y_P/S_ by 50% (Fig. 3A). Under the latter condition 9.7 ± 0.5 mM, succinate was produced by *V. natriegens* WT at a yield Y_P/S_ = 0.38 ± 0.02 mol_Succ_ mol_Glc_^−1^. The effect of KHCO_3_ supplementation remained marginal, when anaerobic tubes were inoculated from stationary precultures. Consequently, we performed all further experiments under the optimized cultivation conditions (inoculum from growing precultures, supplementation of 100 mM KHCO_3_).

### Engineering *V. natriegens* for succinate production

**Fig. 4:**
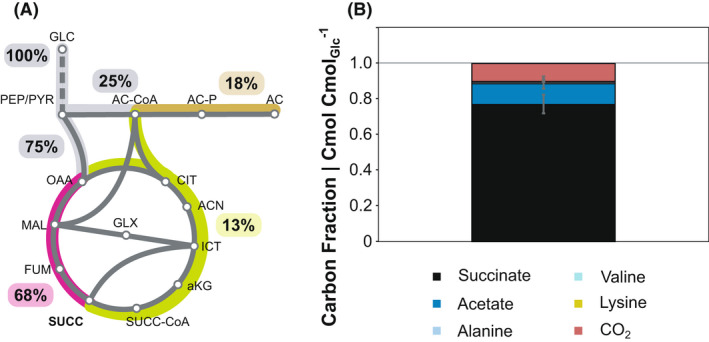
Metabolic flux distribution (A) and refined carbon balance (B) of *V. natriegens* Succ1 (∆*lldh* ∆*dldh* ∆*pfl* ∆*ald* ∆*dns*::*pyc*_Cg_). As outlined above, we could not distinguish between carbon flux via the oxidative branch of the TCA cycle and glyoxylate pathway (A). For simplicity, only the oxTCA route is highlighted.

To improve the precursor availability for succinate production and to reduce by‐product formation, we engineered *V. natriegens* by inactivating competing pathways. *V. natriegens* Δ*lldh* Δ*dldh* Δ*pfl* has been constructed recently to abolish/mitigate lactate, acetate, formate and ethanol formation (Hoffart *et al*., 2017) and showed here under the tested conditions a Y_P/S_ of 0.90 ± 0.11 mol_Succ_ mol_Glc_^−1^ (Fig. 3B). Since this strain secreted 0.71 ± 0.08 mol alanine per mol glucose (Fig. 3C), we additionally deleted the *ald* gene annotated as alanine dehydrogenase, which potentially regenerates NAD^+^ by the reductive amination of pyruvate under anaerobic conditions. The resulting strain *V. natriegens* Δ*lldh* Δ*dldh* Δ*pfl* Δ*ald* showed significantly reduced alanine formation (0.07 ± 0.01 mol_Ala_ mol_Glc_^−1^); however, the Y_P/S_ was only slightly improved to 1.01 ± 0.08 mol_Succ_ mol_Glc_^−1^ (Fig. 3B and C). Instead, 30% of glucose‐derived carbon was directed to another so far unknown by‐product (Fig. 3C), which could not be detected as amino or organic acid (including pyruvate) in the supernatant. All deletions made in *V. natriegens* Δ*lldh* Δ*dldh* Δ*pfl* Δ*ald* led to a potentially elevated intracellular pyruvate availability. However, *V. natriegens* does not possess a pyruvate carboxylase to support the anaplerotic flux from pyruvate. To account for the possibility that PEP synthase activity, catalysing the pyruvate conversion to PEP, or the subsequent carboxylation was insufficient in *V. natriegens*, we chromosomally integrated the *pyc* gene encoding pyruvate carboxylase from *C. glutamicum* at the *dns* gene locus (Weinstock *et al*., 2016; Dalia *et al*., 2017). Pyc catalyses the carboxylation of pyruvate to produce oxaloacetate, thus enhancing the anaplerotic flux (Peters‐Wendisch *et al*., 1997, 1998). The resulting strain *V. natriegens* Δ*lldh* Δ*dldh* Δ*pfl* Δ*ald* Δ*dns*::*pyc*_Cg_ (Succ1) yielded 1.46 ± 0.08 mol_Succ_ mol_Glc_^−1^ (Fig. 3B), which represents 85% of the theoretical maximum, and indicates that under our test conditions the oxidative branch of the TCA cycle and/or the glyoxylate pathway actively contributed to succinate formation. Acetate was secreted as the main by‐product (0.38 ± 0.08 mol_Ac_ mol_Glc_^−1^), accounting for 12% of the glucose‐derived carbon (Fig. 3C), and the combined yield of amino acids (alanine, valine and lysine) summed up to 0.02 ± 0.01 mol mol_Glc_^−1^ in the final succinate‐producing strain. Another 12% of the glucose‐derived carbon could not be quantified. A degree of reduction balance was generally in good agreement with the results of the carbon balance (Fig. S2, Table S2). Interestingly, for *V. natriegens* Succ1, the degree of reduction balance was closed to 100%, suggesting that the missing carbon of the carbon balance is redox neutral (Fig. S2, Table S2). It became evident that the missing carbon sink was CO_2_, when we estimated the metabolic flux distribution by feeding the experimental data into the reaction equations (1)–(3) and balancing reduction equivalents as before (Fig. 4). The glucose‐derived carbon was mainly routed towards succinate via the redTCA (68%), acetate (18%) and 13% contributed to succinate formation via the oxTCA (Fig. 4A). Eventually, we refined the preliminary carbon balance, with the knowledge about the intracellular flux distribution (Fig. 4B). The theoretical CO_2_ release within the oxTCA accounted for 10% of the missing carbon. Another 2% of the missing carbon was identified as succinate, which was produced via the oxTCA. Unlike succinate produced via the redTCA (3 glucose‐derived carbon atoms), this fraction contains four glucose‐derived carbon atoms (Fig. S1). By calculation of the intracellular flux distribution, we could close the previous gap of the carbon balance to 100% (Fig. 4B), indicating that there was no undetected by‐product formed by *V. natriegens* Succ1.

*Vibrio natriegens* Succ1 grew in minimal medium containing glucose as sole carbon and energy source without a lag phase at µ = 1.41 ± 0.02 h^−1^, which represents 90% of the WT in the same experiment (µ = 1.57 ± 0.08 h^−1^, Fig. S3). Anaerobically resting cells of *V. natriegens* Succ1 consumed glucose at q_S_ = 1.38 ± 0.19 g_Glc_ g_CDW_^−1^ h^−1^ within 3 h and secreted succinate at q_P_ = 1.33 ± 0.18 g_Succ_ g_CDW_^−1^ h^−1^ within the first 2 h (Fig. 3D).

Last, we attempted to increase the succinate yield through the mitigation of acetate formation, which might be catalysed by the phosphate acetyltransferase and acetate kinase. Both enzymes are annotated by two independent genes, respectively. The deletion of the *pta‐ackA* operon on chromosome 1 in *V. natriegens* Succ1 resulted in a strain, that did no longer consume glucose as sole carbon and energy source under aerobic as well as anaerobic conditions although it grew well in complex medium. The deletion of both *ackA* genes in the same background remained without effect and produced the same amount of acetate as *V. natriegens* Succ1 (data not shown).

### Zero‐growth anaerobic bioprocess

**Fig. 5:**
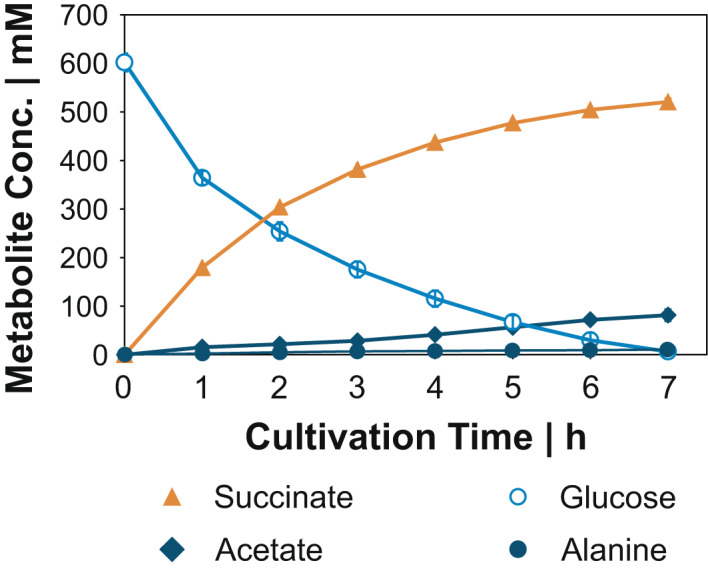
Anaerobic zero‐growth succinate production with *V. natriegens* Succ1 (∆*lldh* ∆*dldh* ∆*pfl* ∆*ald* ∆*dns*::*pyc*_Cg_). The bioreactor system contained initially 400 ml VN minimal medium with 555 mM glucose and 150 mM KHCO_3_. 100 mM KHCO_3_ h^−1^ was continuously fed into the bioreactor. All data represent mean values with error bars indicating the standard deviation of three independent biological replicates.

Finally, we scaled up the anaerobic resting cell approach with *V. natriegens* Succ1 to a lab‐scale bioreactor, which we inoculated to a starting biomass concentration of 18 g l^−1^. We used VN minimal medium lacking any complex additives and expensive nutrients. The anaerobic process was highly reproducible and reached within 7 h a final succinate titre of 520.7 ± 7.7 mM (i.e. 60.4 g l^−1^) from glucose and KHCO_3_ which corresponds to a Y_P/S_ of 1.14 ± 0.02 mol_Succ_ mol_Glc_^−1^. Y_P/S_ remained constant during the entire fermentation. Acetate was the major by‐product with a yield of 0.18 ± 0.02 mol_Ac_ mol_Glc_^−1^, followed by alanine with a yield of 0.02 mol_Ala_ mol_Glc_^−1^ (Fig. 5). Considering these products, 64% of the initial glucose was recovered by carbon balancing and 73% by a degree of reduction balance. Other carbon sinks (including organic acids, amino acids and alcohols, which we analysed routinely) were not detected, indicating a considerable fraction of unknown products. The biomass‐specific productivity started at levels, that were almost as high as in the small anaerobic cultivation system (1.15 ± 0.8 g_Succ_ g_CDW_^−1^ h^−1^), and decreased with the overall process performance. The anaerobic process achieved an overall volumetric productivity of 74.4 ± 1.1 mmol succinate l^−1^ h^−1^ (i.e. 8.6 ± 0.1 g l^−1^ h^−1^). During the first hour of the process with *V. natriegens* Succ1, the maximum volumetric productivity of 179.3 ± 1.9 mmol succinate l^−1^ h^−1^ (i.e. 20.8 ± 0.2 g l^−1^ h^−1^) was reached.

## Discussion

In this study, we increased the succinate yield of *V. natriegens* 10‐fold through optimization of the anaerobic cultivation conditions and applying a minimal metabolic engineering approach. Under optimized conditions, the Y_P/S_ of *V. natriegens* WT reached about 55% of the designated succinate producer *M. succiniciproducens* WT (Lee *et al*., 2006). The final production strain *V. natriegens* Succ1 maintained wild type‐like fitness under aerobic conditions (growth rate reduced by only 10% compared with the WT, Fig. S3) and yielded 1.46 ± 0.08 mol_Succ_ mol_Glc_^−1^, which represents 85% of the theoretical maximum with glucose as sole organic substrate. Particularly, the high yield and biomass‐specific productivity of 1.33 ± 0.18 g_Succ_ g_CDW_^−1^ h^−1^ place *V. natriegens* Succ1 amongst the best performing succinate producers reported so far (Ahn *et al*., 2016, 2020; Kim *et al*., 2017; Lee *et al*., 2019b; Yang *et al*., 2020; Dai *et al*., 2020). Carbon and degree of reduction balancing indicated that in a small‐scale cultivation of *V. natriegens* Succ1 acetate was the only major by‐product beside CO_2_. We calculated the intracellular carbon flux distribution under these conditions by feeding a metabolic network with the experimental data (glucose, succinate and acetate concentrations). Accordingly, succinate was produced by both pathways – redTCA and oxTCA (or glyoxylate pathway).

The very high biomass‐specific productivity of *V. natriegens* Succ1 could finally be translated into an exceptionally high overall volumetric productivity (8.6 g_Succ_ l^−1^ h^−1^ during the production phase), when we performed the production under pH‐controlled bioreactor conditions. Within only 7 h, we achieved final succinate titres that compete with commonly reported literature values (Table 1). However, higher titres have been achieved with various organisms under optimized fermentation conditions (Ahn *et al*., 2016; Lee *et al*., 2019b; Yang *et al*., 2020; Dai *et al*., 2020) and must be improved significantly with *V. natriegens* Succ1 in the future, to facilitate downstream processing. Our maximum volumetric productivity during the initial production phase (20.8 g_Succ_ l^−1^ h^−1^) is at the same level as a recently developed process using engineered *M. succiniciproducens* of 20.9 g_Succ_ l^−1^ h^−1^ (= 21.3 g succinic acid l^−1^ h^−1^) in an identical set‐up (Ahn *et al*., 2020). In contrast to the latter and many other studies (Vemuri *et al*., 2002a; Sánchez *et al*., 2005; Zhu *et al*., 2014; Lee *et al*., 2019b; Ahn *et al*., 2020; Dai *et al*., 2020), our production process did not require the addition of vitamins, amino acids and other expensive supplements. The succinate yield of the bioreactor cultivation was significantly lower than that of the small test system. Carbon and degree of reduction balancing suggested a considerable proportion of glucose was converted to a reduced product. However, we could not identify the carbon sink in this set‐up, as about 50% less acetate was formed in the larger set‐up and no other organic acids, alcohols or amino acids were detected, except for alanine that made up about 1% of the glucose‐derived carbon.

**Table 1:** Selected anaerobic succinate production processes with genetically modified organisms in fed‐batch mode (^a^ yield calculated as mol succinate per mol glucose equivalent (carbon basis), maximum theoretical yield for the given set‐up (considering substrates + reduction degree) without consideration of complex compounds and organic supplements; ^b^ calculated from data for succinic acid).

Organism	Substrate(s)	Complex/organic supplements	Volumetric productivity (maximum productivity)	Biomass‐specific productivity (maximum)	Final succinate titre	Yield^a^ (% of theoretical maximum yield)	Duration	Reference
			g_Succ_ l^−1^ h^−1^	g_Succ_ g_CDW_^−1^ h^−1^	g l^−1^	mol_Suc_ mol_Glc_^−1^	h	
*Vibrio natriegens* ∆*lldh* ∆*dldh* ∆*pfl* ∆*ald* ∆*dns*::*pyc*_Cg_	Glucose + KHCO_3_	–	8.6 (20.8)	0.48 (1.15)	60.4	1.14 (67%)	7	This study
*Actinobacillus succinogenes* NJ113	Sucrose + CO_2_ + Na_2_CO_3_ (base titration)	Yeast extract + corn steep liquor	2.1 (–)^b^	–	59.5^b^	1.12^b^ (66%)	28	Jiang *et al*. (2014)
*Corynebacterium glutamicum* ∆*ldhA* (pCRA717)	Glucose + HCO_3_^‐^	Biotin + thiamin	3.2 (11.6)^b^	0.06 (0.23)^b^	143.6^b^	1.40 (82%)	46	Okino *et al*. (2008)
*Corynebacterium glutamicum* BOL‐3 (pAN6*‐gap*)	Glucose + formate + NaHCO_3_	–	2.53 (–)	0.19 (–)	133.8	1.67 (84%)	53	Litsanov *et al*. (2012)
*Escherichia coli* AFP111 (pTrc99A‐*pyc*)	Glucose + CO_2_ + Na_2_CO_3_ (base titration)	Yeast extract + tryptone + biotin + thiamin	1.3 (–)	0.13 (–)	97.5	1.78 (105%)	75	Vemuri *et al*. (2002b)
*Mannheimia succiniciproducens* PALK cgmdh	Glucose + glycerol + NaHCO_3_ + CO_2_	Biotin, Ca‐pantothenate, pyridoxine‐HCl, thiamine, ascorbic acid nicotinic acid + amino acids (Ala, Asn, Asp, Cys, Met, Pro, Ser)	10.2 (20.9)^b^	1.17 (2.40)^b^	132^b^	1.25 (71%)	13	Ahn *et al*. (2020)

Consequently, future work is essential to elucidate the physiological response of *V. natriegens* to the bioreactor environment. The re‐direction of carbon fluxes might be triggered by *quorum sensing* stimuli at high cell densities (Thoma and Blombach, 2021). Process intensification strategies are needed to increase titre and yield further and enable industrially relevant dual‐phase fermentations by combining biomass and product formation in a single vessel and using only minimal media (Lange *et al*., 2016; Kumar *et al*., 2020; Raj *et al*., 2020). The development of such approaches is challenging since the physiological state of the cells entering the transition from aerobic to anaerobic conditions can significantly impact product formation as shown for succinate production with *E. coli* (Vemuri *et al*., 2002b; Martínez *et al*., 2010). Thus, system‐level analysis in combination with process optimization will probably be required also for *V. natriegens* to optimally adjust the enzymatic machinery for the anaerobic production phase.

Interestingly, the succinate yield of *V. natriegens* WT was higher, when the production was started with a growing preculture instead of cells from the stationary phase. This suggests a severe reduction of enzyme activity contributing to the anaplerotic flux or downstream reactions of the reductive TCA branch when cells transition to stationary phase. Consequently, and in contrast to growing precultures, the effect of KHCO_3_ supplementation remained marginal when we used stationary precultures. The anaplerotic flux in *V*. *natriegens* is provided by PEP carboxylation under aerobic conditions (Long *et al*., 2017), which should be catalysed primarily by the PEP carboxylase (Sauer and Eikmanns, 2005). However, a contribution of the reversible PEP carboxykinase to the C3 carboxylation has been demonstrated in various organisms (Sauer and Eikmanns, 2005) and the enzyme is responsible for about 90% of the anaplerotic carbon flux in *M*. *succiniciproducens* during succinate production under anaerobic conditions (Lee *et al*., 2006). A pyruvate carboxylase gene is not encoded in the genome of *V. natriegens* and pyruvate accumulates frequently, when the oxygen demand is insufficient (Thoma and Blombach, 2021). Therefore, the positive effect of *pyc* overexpression on the succinate yield was consistent with the outcome of similar engineering strategies that have been applied to other host organisms in the past (Vemuri *et al*., 2002a).

Our metabolic engineering efforts to mitigate acetate formation revealed some interesting insights in the physiological peculiarities of *V. natriegens*. In the Succ1 strain background, acetate seems to be formed from acetyl‐CoA, since a gene encoding a pyruvate oxidase is not annotated in the genome. In accordance with Lee *et al*., who showed that both genes of the *pta‐ackA* operon on chromosome 1 are essential in the wild type during aerobic growth on glucose (Lee *et al*., 2019a), *V. natriegens* Succ1 ∆(*pta‐ackA*) did not consume glucose as sole carbon and energy source. Apparently, neither the gene duplicates on chromosome 2 nor other annotated CoA‐transferases or acetaldehyde/alcohol dehydrogenases, that could theoretically catalyse the same reaction, compensated the deletion of the *pta‐ackA* operon in this strain background. In this regard, *V. natriegens* differs from other organisms, in which the disruption of the *pta‐ackA* pathway was a successful engineering approach to abolish or reduce acetate formation under anaerobic conditions (Sánchez *et al*., 2005; Lee *et al*., 2006; Litsanov *et al*., 2012). The deletion of both annotated *ackA* genes in *V. natriegens* Succ1 remained without effect on the acetate yield. Altogether these results suggest, that acetyl phosphate is exclusively formed by the phosphate acetyltransferase (encoded on chromosome 1) and might play an indispensable physiological role in *V. natriegens*. The acetate generation from acetyl phosphate might be catalysed by the acylphosphatase, if both acetate kinase genes are deleted. The corresponding gene is annotated in the genome of *V. natriegens* (Long *et al*., 2017).

## Conclusion

Here, we present the first approach to engineer *V. natriegens* for the biotechnological production of succinate with remarkable key performance indicators. Scope for improvement remains particularly in the process intensification and scale‐up. Especially, the final titre needs to be increased to realize cost‐efficient purification of succinate. Future studies have to develop sophisticated dual‐phase processes to combine the high growth rate and metabolic activity for rapid biomass and product formation in a single industrially relevant bioprocess. However, the high biomass‐specific productivity of our engineered strain in cheap minimal medium devoid of expensive supplements sets already a benchmark for succinate production with *V. natriegens*.

## Experimental procedures

### Bacterial strains, plasmids and cultivation media

An overview of the bacterial strains and plasmids utilized during this study is provided in Table 2. Permanent cultures of all strains were maintained at −80 °C in 30% (v v^−1^) glycerol. *E. coli* S17‐1 λ*pir* cells were cultivated in 2× YT medium containing per l: 16 g Bacto tryptone, 10 g yeast extract and 5 g NaCl (Sambrook and Russell, 2001). *V. natriegens* strains were precultured in 2x YTN containing an elevated concentration (15 g l^−1^) of NaCl and 50 mM MgCl_2_. Main cultures (growth experiments and succinate production) were set up in VN minimal medium containing per l: 5 g (NH_4_)_2_SO_4_, 21 g 3‐(*N*‐morpholino)propanesulfonic acid (MOPS), 1 g KH_2_PO_4_, 1 g K_2_HPO_4_, 15 g NaCl, 10 mg CaCl_2_ ∙ H_2_O, 250 mg MgSO_4_ ∙ 7 H_2_O, 16.4 mg FeSO_4_ ∙ 7 H_2_O, 10 mg MnSO_4_ ∙ H_2_O, 1 mg ZnSO_4_ ∙ 7 H_2_O, 0.3 mg CuSO_4_ ∙ 5 H_2_O, 0.02 mg NiCl_2_ ∙ 6 H_2_O (Hoffart *et al*., 2017). The medium pH was adjusted to 7.5 with 5 M KOH. The indicated amount of glucose and KHCO_3_ was added aseptically. When appropriate, 15 µg chloramphenicol ml^−1^ was used. MOPS buffer was omitted from media, when pH‐controlled bioreactor cultivations were performed.

**Table 2:** Bacterial strains and plasmids used in this study.

Strain / plasmid	Relevant characteristics	Source / reference
*E. coli* S17‐1 λ*pir*	*thi pro hsdR hsdM1 recA* RP4‐2‐Tc::Mu‐Km::Tn7 λ*pir*	Simon *et al*. (1983)
*V. natriegens*	wild type (WT) strain DSM 759 (ATCC 14048)	German Collection of Microorganisms and Cell Cultures
*V. natriegens* Δ*lldh* Δ*dldh* Δ*pfl*	*V. natriegens* WT with deletion of the *lldh, dldh,* and *pfl* genes encoding d‐lactate dehydrogenase, l‐lactate dehydrogenase and pyruvate formate lyase (locus tags: PN96_16785, PN96_16800 and PN96_08455)	Hoffart *et al*. (2017)
*V. natriegens* Δ*lldh* Δ*dldh* Δ*pfl* Δ*ald*	*V. natriegens* Δ*lldh* Δ*dldh* Δ*pfl* with additional deletion of the *ald* gene encoding alanine dehydrogenase (locus tag: PN96_09745)	This work
*V. natriegens* Δ*lldh* Δ*dldh* Δ*pfl* Δ*ald* Δ*dns*::*pyc*_Cg_ (= Succ1)	*V. natriegens* Δ*lldh* Δ*dldh* Δ*pfl* Δ*ald* with genomic integration of the *pyc* gene from *C. glutamicum* ATCC 13032 encoding pyruvate carboxylase into the *dns* gene (locus tag: PN96_00865)	This work
*V. natriegens* Succ1 Δ(*pta*‐*ackA*)	*V. natriegens* Succ1 with additional deletion of the *pta*‐*ackA* operon encoding phosphate acetyltransferase and acetate kinase (locus tag: PN96_03360‐65)	This work
*V. natriegens* Succ1 Δ*ackA*1 Δ*ackA*2	*V. natriegens* Succ1 with additional deletion of both *ackA* genes encoding acetate kinase (locus tags: PN96_03365 and PN96_21510)	This work
*C. glutamicum*	ATCC 13032	American Type Culture Collection, Abe *et al*. (1967)
pDM4	*oriR6K*, *oriT*, *sacB,* Cm^R^	Milton *et al*. (1996)
pDM4‐Δ*ald*	pDM4 derivative carrying 500 bp homologous sequences for the deletion of the *ald* gene	This work
pDM4‐Δ*dns*::*pyc*_Cg_	pDM4 plasmid with an integration construct of the *pyc* gene from *C. glutamicum* under control of the *tac* promoter and followed by the *rrnB* terminator; *dns* gene is disrupted	This work
pDM4‐Δ(*pta*‐*ackA*)	pDM4 derivative carrying 500 bp homologous sequences for the deletion of the *pta‐ackA* operon	This work
pDM4‐Δ*ackA*1	pDM4 derivative carrying 500 bp homologous sequences for the deletion of the *ackA*1 gene	This work
pDM4‐Δ*ackA*2	pDM4 derivative carrying 500 bp homologous sequences for the deletion of the *ackA*2 gene	This work
pJC4‐P_tac_‐*egfp*	*oriV*_E. coli_, pCG1 *oriV_C. glutamicum_*, Kan^R^, for *egfp* expression under *tac* promoter, followed by the *rrnB* terminator	Müller *et al*. (2020)

### Genetic manipulation

Standard techniques of molecular biology (e.g. agarose gel electrophoresis and PCR amplification) were applied as described in the literature (Sambrook and Russell, 2001). Chromosomal DNA and plasmid DNA were isolated using the NucleoSpin Microbial DNA and the NucleoSpin Plasmid kit (Macherey‐Nagel, Düren, Germany) respectively. PCR fragments were purified with the NucleoSpin Gel and PCR Clean‐up kit (Macherey‐Nagel) according to the manufacturer’s instructions. Enzymes were purchased from New England Biolabs (Frankfurt, Germany), and oligonucleotides were ordered from Sigma‐Aldrich (Steinheim, Germany). Chromosomal modifications in *V. natriegens* were made using pDM4‐derived plasmids allowing markerless integration and deletion by selection on chloramphenicol and *sacB*‐based counter‐selection (Milton *et al*., 1996). For the deletion, 500 bp sequences flanking the *ald* gene (PN96_07945) were PCR amplified with the primer pairs ald1/ald2 and ald3/ald4, respectively (Table S1) and simultaneously inserted into the XbaI‐ and SalI‐linearized pDM4 plasmid by isothermal assembling (Gibson, 2011). Accordingly, homologous sequences for the deletion of the *pta‐ackA* operon (PN96_03360‐65), the *ackA1* gene (PN96_03365) and the *ackA2* gene (PN96_21510) were PCR amplified with the primers as indicated in Table S1. Chromosomal integration of the *pyc* gene was targeted to the *dns* gene locus (PN96_00865) by amplifying 500 bp sequences with the primer pairs dns1/dns2 and dns3/dns4, and the expression module P_tac_‐*pyc*‐T_rrnB_ was placed in between the two flanks. The *pyc* gene was amplified from the chromosomal DNA of *C. glutamicum* ATCC 13032 using the primer pair pyc1/pyc2. The native GTG start codon was replaced with the stronger ATG during the amplification step with a primer encoded point mutation. Promoter and terminator elements were amplified from pJC4‐P_tac_‐*egfp* (Müller *et al*., 2020) using Ptac1/Ptac2 and TrrnB1/TrrnB2 respectively. All fragments were simultaneously assembled with the XbaI‐ and SalI‐linearized pDM4 plasmid as described before.

Competent cells of *E. coli* S17‐1 λ*pir* were transformed with the resulting plasmids by electroporation following the protocol described by (Lange *et al*., 2018). Plasmids were amplified and isolated, and the insert was verified by sequencing (Eurofins, Constance, Germany) before conjugating it into *V. natriegens* as described previously (Hoffart *et al*., 2017). We used 2x YTN medium during the conjugation protocol instead of BHIN. Insertion/deletion mutants were generated by two homologous recombination steps (Milton *et al*., 1996) and eventually verified by colony PCR.

### Small‐scale cultivation experiments

The previously established seed train (Hoffart *et al*., 2017) was slightly modified in this study by replacing BHIN medium with 2× YTN medium. Experiments were started by streaking out cells from cryogenic cultures (−80 °C) on 2× YTN agar plates, which were incubated for 6–8 h or overnight (O/N) at 37 °C. 5 ml liquid 2× YTN medium in test tubes was inoculated with a single colony from the plate and incubated O/N at 37 °C shaking (180 rpm), before cells were pelleted by centrifugation (10 min, room temperature (RT), 4347 *g*). For aerobic shaking flask experiments, the cell pellet was re‐suspended in 0.9% (w v^−1^) NaCl and the optical density at 600 nm (OD_600_) of the suspension was adjusted, in order to inoculate the main culture (VN minimal medium) to a starting biomass concentration of 26 mg_CDW_ l^−1^ (OD_600_ = 0.1). Experiments were performed in 500 ml baffled shaking flasks containing 50 ml VN medium and 55 mM glucose as sole carbon and energy source (Hoffart *et al*., 2017).

Routinely, anaerobic succinate production was performed in 50 ml test tubes with screw caps (to prevent aeration during the experiment) containing 50 ml VN minimal medium with 27.5 mM glucose as sole organic carbon and energy source and 100 mM KHCO_3_ as inorganic carbon source to provide the essential substrate for the anaplerotic carboxylation reactions. With the biomass‐specific oxygen consumption rate (28 mmol_O2_ g_CDW_^−1^ h^−1^, Thoma and Blombach, 2021) the residual oxygen present in 50 ml liquid phase and 5 ml gas of the head space was exhausted after approximately 34 s, and strictly anaerobic conditions established thereafter. The initial glucose concentration was lower in anaerobic experiments, in order to reduce the medium acidification through organic acid secretion. Deviations from this procedure are stated in the results section. For growing precultures, a 50 ml 2× YTN preculture (in a 500 ml baffled shaking flask) was inoculated with 500 µL of the O/N culture and incubated at 37 °C until an OD_600_ of about 5 was reached. Cells were harvested as before, re‐suspended, and anaerobic tubes were inoculated with an initial biomass concentration of 1.3 g_CDW_ l^−1^ (OD_600_ = 5). The inoculum from stationary phase cells was taken from 5 ml O/N cultures straight away and processed likewise.

### Two‐stage anaerobic bioprocess

The final anaerobic succinate production processes were performed in 2 l glass vessels of a DASGIP bioreactor system (Jülich, Germany). To obtain enough biomass, precultures were set up in 3× 1.5 l 2× YTN medium and incubated at 37 °C until an OD_600_ of about 5 was reached (after 2.5 h cultivation). The bioreactors were aerated at 0.5 vvm with pressurized air and stirred at 600 rpm (with three Rushton turbines) to achieve microaerobic conditions during the cultivation. The dissolved oxygen concentration was detected with a polarographic probe (Mettler Toledo, Giessen, Germany). Precultures were harvested by centrifugation (10 min, RT, 4347 *g*), and the cell pellet was used to inoculate 400 ml VN medium to a starting biomass concentration of about 18 g_CDW_ l^−1^. VN medium initially contained 555 mM glucose and 150 mM KHCO_3_. For succinate production, bioreactors were not aerated and the stirrer speed was set to 180 rpm. The culture pH was monitored with a standard pH probe (405‐DPAS‐SC‐K8S/325, Mettler Toledo, Giessen, Germany) and maintained at 7.5 by a two‐side regulation using 13.3 M NH_4_OH and 1.1 M H_3_PO_4_. A linear KHCO_3_ feed was applied at 100 mmol l^−1^ h^−1^.

### Analytics

#### Determination of growth parameters

The biomass concentration (c_CDW_) was monitored by measuring the optical density at 600 nm (OD_600_). The correlation factor to convert c_CDW_ to g_CDW_ l^−1^ had been determined from exponentially growing cells for the spectrophotometer (Ultrospec 10 cell density meter, Harvard Biochrom, Holliston, MA, USA): c_CDW_ = OD_600_ × 0.26. The growth rate (µ) was determined in the exponential phase through a regression curve, fitting the slope of the biomass concentration in a semilogarithmic plot over time.

#### Quantification of sugars, organic acids and alcohols

Glucose, succinate, pyruvate, acetate, lactate, formate and ethanol were quantified by high‐performance liquid chromatography (HPLC) using an Agilent 1260 infinity II series device (Agilent Technologies, Waldbronn, Germany) equipped with a Hi‐Plex H column (7.7 × 300 mm, 8 µm) and Hi‐Plex H guard cartridge (3.0 × 5.0 mm, 8 µm) for protection. Analytes were eluted at 50 °C isocratically using 5 mM H_2_SO_4_ (mobile phase, flow = 0.4 ml min^−1^) and detected with a refractive index detector (RID). Analyte concentrations were calculated by comparing the peak area of the sample with the peak area of a 7‐point external standard ranging from 1 to 200 mM (Siebert *et al*., 2021).

#### Quantification of amino acids

Amino acids were quantified by HPLC using an identical device as described before equipped with an AdvanceBio Amino Acid Analysis (AAA) column (4.6 × 100 mm, 2.7 µm) and AdvanceBio AAA guard column (4.6 × 5 mm, 2.7 µm) for protection. Primary amino acids were online derivatized with *o*‐phthaldialdehyde (OPA) prior to separation through a gradient of polar (10 mM Na_2_HPO_4_, 10 mM Na_2_B_4_O_7_, pH 8.2) versus non‐polar phase (45% v v^−1^ acetonitrile, 45% v v^−1^ methanol and 10% v v^−1^ water) by increasing the linear gradient stepwise from 2–43% non‐polar fraction within 10 min and 43–100% within 2:30 min, at a total flow rate of 1 ml min^−1^, 40 °C. The analytes were detected with a fluorescence detector (FLD, excitation wavelength: 340 nm, emission wavelength: 450 nm and PMT gain: 10). Peak areas were normalized to the internal standard (100 µM norvaline), and concentrations were finally calculated by comparing the peak area of the sample with the peak area of a 7‐point external standard ranging from 1 to 400 µM (Siebert *et al*., 2021).

### Statistical analysis

All experiments were performed at least in independent biological triplicate on different days. Seed trains of all replicates were started by streaking cell material of a cryogenic culture on a fresh agar plate. Data represent mean values of at least triplicate, and error bars indicate standard deviations. If appropriate, standard deviations were calculated by error propagation.

#### Carbon and degree of reduction balancing

The carbon balance for the anaerobic resting cell approach was calculated on the C‐molar basis in accordance with Buchholz *et al*. (2014). C‐molar concentrations of the HPLC analytes were summed up, and the individual fraction deriving from the initial glucose was determined. Due to the lack of biomass formation in the resting cell approach, it was not considered in the carbon balance. According to the succinate production mode, we assumed initially that succinate was produced via the reductive branch of the TCA cycle. Consequently, only three carbon atoms of the C4‐molecule were considered as ‘glucose‐derived’. The gap between products and substrates was designated as ‘unknown’. All standard deviations were calculated by error propagation of the biological triplicate. The carbon balance of *V. natriegens* Succ1 was refined after the estimation of carbon fluxes for succinate production. Four carbon atoms of succinate were considered as glucose‐derived, when it was produced via the oxidative arm of the TCA cycle (considerations are visualized in Fig. S1). CO_2_ release was not quantified, but integrated in the theoretical considerations, when flux passed the decarboxylation reactions (pyruvate dehydrogenase complex (PDHC), isocitrate dehydrogenase and α‐ketoglutarate dehydrogenase complex).

In addition to this, a degree of reduction balance was set up, in order to account for the individual contribution of each product independently of CO_2_/HCO_3_^‐^ evolution. In accordance with Villadsen *et al*. (2011), redox levels of all quantified metabolites (glucose = 24, succinic acid = 14, acetic acid = 8, alanine = 12, ethanol = 12, formic acid = 2, lactic acid = 12, valine = 24 and lysine = 28) and the reduction degrees (κ_i_) per C‐mole were calculated (Villadsen *et al*., 2011). The redox contribution of each product was calculated through multiplying κ_i_ by the C‐molar concentration. All products were summed up and expressed as fraction of glucose, in analogy with the carbon balance.

#### Metabolic flux analysis

A metabolic network of all relevant fluxes for succinate and acetate (as the most prominent by‐product of succinate formation) production from glucose in *V. natriegens* Succ1 was set up in accordance with the genome annotation (ATCC 14048) and KEGG database (Kanehisa and Goto, 2000; Lee *et al*., 2019a). The network comprises glycolysis, acetate metabolism (considering only the Pta‐AckA and Acs catalysed reactions in *V. natriegens* Succ1), reductive branch of the TCA cycle (redTCA), oxidative branch of the TCA cycle (oxTCA) and glyoxylate pathway (Fig. 2). Based on the overall reactions of these modules, equations (1)–(3) describe the conversion of glucose to either succinate or acetate via a combination of glycolysis and redTCA (1), glycolysis and oxTCA/ or glyoxylate pathway (2), and converting glucose to acetate (3).

(1) $$‐\text{Glc}‐2\;{\text{CO}}_{2}‐2\;\text{NADH}+2\;\text{Succ}=0$$

(2) $$‐\text{Glc}+2\;{\text{CO}}_{2}+5\;\text{NADH}+\text{Succ}=0$$

(3) $$‐\text{Glc}+2\;{\text{CO}}_{2}+4\;\text{NADH}+2\;\text{Ac}=0$$

The theoretical environment was an anaerobic production scenario with resting cells, premising no biomass generation from glucose and full conservation of reduction equivalents. Reduction equivalents were considered as the aggregate of NAD(P)H and quinones. We assumed that all pathways were fully operative under the given conditions, and reduction equivalents were interconvertible. ATP energetics were not considered in the calculations, as we could not experimentally verify the carbon flux through either of the energetically different reactions (i.e. PEP carboxylase/pyruvate carboxylase vs. PEP carboxykinase). The optimum flux distribution for a maximum succinate yield from glucose was calculated by balancing the consumption/production of reduction equivalents (1) and (2). In addition, the maximum yield was calculated in a second scenario considering a potentially inactive oxTCA under anaerobic conditions (contribution of (2) = 0).

The metabolic flux distribution during the small‐scale experiment (Succ1) was estimated by feeding experimental data of glucose, succinate and acetate quantification into equations (1)–(3) and balancing the reduction equivalents as before. Only mean values of the analytes were used.

## Author contributions

BB acquired funding of the project. FT, CS and BB conceived and designed the experiments. FT and RT performed the metabolic modelling. FT, CS, CGC, CG, JH and MH performed the experiments and analysed the data. FT and BB drafted the manuscript. All authors reviewed and agreed with the final manuscript.

## Conflict of interest

The authors declare that there are no competing interests associated with this work.

## Supporting information


**Fig. S1**. Illustration of the succinate formation and its discrepancy regarding the origin of carbon atoms. Depending on the production pathway, all four carbon atoms of succinate originate from glucose (oxidative branch of the TCA cycle, green). Alternatively, only three carbon atoms of succinate originate from glucose and the fourth carbon atom is provided by CO_2_/HCO_3_ ‐ during the carboxylation of PEP and/or pyruvate (reductive branch of the TCA cycle, magenta). With regards to their origin, glucose‐derived carbon atoms are shown as black circles, whereas CO_2_/HCO_3_ ‐ derived carbon atoms are highlighted as yellow circles.
**Fig. S2**. Degree of reduction balance of anaerobic succinate production in test tubes containing 50 mL VN minimal medium with 27.5 mM glucose. Reduction degree times C‐molar concentration (κi * ci) of each product (i) is described as fraction of glucose (κGlc * cGlc)^−1^.
**Fig. S3**. Aerobic shaking flask cultivations of *V. natriegens* WT and *V. natriegens* Succ1 in VN minimal medium containing 10 g glucose l^−1^. Data points represent the mean and error bars indicate the standard deviation of three independent biologicals replicates.
**Table S1**. Oligonucleotides used in this study.
**Table S2**. Comparison of the carbon fraction recovered by balancing carbon atoms and degree of reduction.
