# Supplementary material for: Metabolic engineering of Vibrio natriegens for anaerobic succinate production
Source: Microb Biotechnol. 2021 Nov 29;15(6):1671–84. doi: 10.1111/1751-7915.13983 (PMC9151343; doi:10.1111/1751-7915.13983)
Supplement: Supplementary file 1 — Fig. S1. Illustration of the succinate formation and its discrepancy regarding the origin of carbon atoms. Depending on the production pathway, all four carbon atoms of succinate originate from glucose (oxidative branch of the TCA cycle, green). Alternatively, only three carbon atoms of succinate originate from glucose and the fourth carbon atom is provided by CO2/HCO3 ‐ during the carboxylation of PEP and/or pyruvate (reductive branch of the TCA cycle, magenta). With regards to their origin, glucose‐derived carbon atoms are shown as black circles, whereas CO2/HCO3 ‐ derived carbon atoms are highlighted as yellow circles. Fig. S2. Degree of reduction balance of anaerobic succinate production in test tubes containing 50 mL VN minimal medium with 27.5 mM glucose. Reduction degree times C‐molar concentration (κi * ci) of each product (i) is described as fraction of glucose (κGlc * cGlc)−1. Fig. S3. Aerobic shaking flask cultivations of V. natriegens WT and V. natriegens Succ1 in VN minimal medium containing 10 g glucose l−1. Data points represent the mean and error bars indicate the standard deviation of three independent biologicals replicates. Table S1. Oligonucleotides used in this study. Table S2. Comparison of the carbon fraction recovered by balancing carbon atoms and degree of reduction. [file MBT2-15-1671-s001.docx]

Supplementary Information

The following information is provided to the article:

**Metabolic engineering of *Vibrio natriegens* for anaerobic succinate production**

In *Microbial Biotechnology*

Felix Thoma^1,2^, Clarissa Schulze^1^, Carolina Gutierrez-Coto^1^, Maurice Hädrich^1^, Janine Huber^1^, Christoph Gunkel^1^, Rebecca Thoma^1^, Bastian Blombach^1,2,*^

^1^Microbial Biotechnology, Campus Straubing for Biotechnology and Sustainability, Technical University of Munich, Straubing, Germany

^2^SynBiofoundry@TUM, Technical University of Munich, Straubing, Germany

^*^Corresponding author: Bastian Blombach (bastian.blombach@tum.de), Microbial Biotechnology, Campus Straubing for Biotechnology and Sustainability, Technical University of Munich, Schulgasse 22, 94315 Straubing, Germany.

**Supplementary Figures**


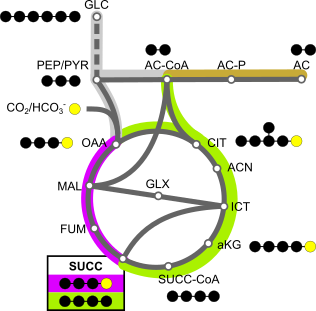


Fig. S1: Illustration of the succinate formation and its discrepancy regarding the origin of carbon atoms. Depending on the production pathway, all four carbon atoms of succinate originate from glucose (oxidative branch of the TCA cycle, green). Alternatively, only three carbon atoms of succinate originate from glucose and the fourth carbon atom is provided by CO_2_/HCO_3_^-^ during the carboxylation of PEP and/or pyruvate (reductive branch of the TCA cycle, magenta). With regards to their origin, glucose-derived carbon atoms are shown as black circles, whereas CO_2_/HCO_3_^-^ derived carbon atoms are highlighted as yellow circles.

**
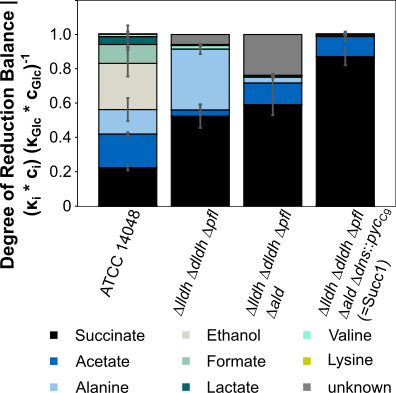
**

Fig. S2: Degree of reduction balance of anaerobic succinate production in test tubes containing 50 mL VN minimal medium with 27.5 mM glucose. Reduction degree times C-molar concentration (κ {\displaystyle \kappa } [κ](https://www.compart.com/de/unicode/U+03BA)_i_ * c_i_) of each product (i) is described as fraction of glucose (κ {\displaystyle \kappa } [κ](https://www.compart.com/de/unicode/U+03BA)_Glc_ * c_Glc_)^-1^.


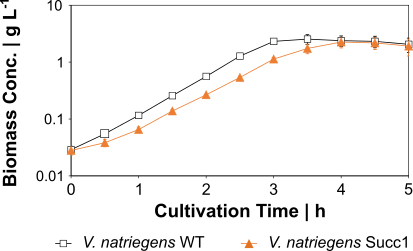


Fig. S3: Aerobic shaking flask cultivations of *V. natriegens* WT and *V. natriegens* Succ1 in VN minimal medium containing 10 g glucose L^-1^. Data points represent the mean and error bars indicate the standard deviation of three independent biologicals replicates.

**Supplementary Tables**

Table S1: Oligonucleotides used in this study

| **Oligonucleotide** | **Sequence (5' → 3')** | **Purpose** |
| --- | --- | --- |
| ald1 | AGTTGAAGTGATGAGATCTATCTAGATAGTCAAAGTCACCTGATACTAG | forward primer for Δ*ald* construct (5'flank) |
| ald2 | TATTTTGTCACCTGTATCCTCTTTCTATAATGG | reverse primer for Δ*ald* construct (5'flank) |
| ald3 | AGGATACAGGTGACAAAATAATTCATTTTTGTCACG | forward primer for Δ*ald* construct (3'flank) |
| ald4 | ATATCAAGCTTATCGATACCGTCGACGAACGTGTCCGCC | reverse primer for Δ*ald* construct (3'flank) |
| dns1 | AGTTGAAGTGATGAGATCTATCTAGAGAGGGATACGCATGAGATTTC | forward primer for *pyc* integration construct targeting the *dns* gene (5'flank) |
| dns2 | GGAAGAGAGTCAATTCAGGGCACAACCTGACTCCTGC | reverse primer for *pyc* integration construct targeting the *dns* gene (5'flank) |
| dns3 | TGTCGGTGAACGCTCTCCTGCAGATCGGCTTCCATTGAG | forward primer for *pyc* integration construct targeting the *dns* gene (3'flank) |
| dns4 | ATATCAAGCTTATCGATACCGTCGACACTGGTGTCCAGCTTTC | reverse primer for *pyc* integration construct targeting the *dns* gene (3'flank) |
| Ptac1 | CCCTGAATTGACTCTCTTCC | forward primer for amplification of the *tac* promoter |
| Ptac2 | ATGGTATATCTCCTTCAATTCTG | reverse primer for amplification of the *tac* promoter |
| pyc1 | AATTGAAGGAGATATACCATATGTCGACTCACACATCTTC | forward primer for amplification of the *pyc* gene |
| pyc2 | ATCCGCCAAAACAGCCAAGCTTAGGAAACGACGACGATC | reverse primer for amplification of the *pyc* gene |
| TrrnB1 | GCTTGGCTGTTTTGGC | forward primer for amplification of the *rrnB* terminator |
| TrrnB2 | CAGGAGAGCGTTCACC | reverse primer for amplification of the *rrnB* terminator |
| pta-ackA1 | AGTTGAAGTGATGAGATCTATCTAGA CTTTACTTACCGGCTTGG | forward primer for Δ(*pta*-*ackA*) construct (5'flank) |
| pta-ackA2 | CGAATAACAGGTAGTCATACTTCTTTTCTCTGCTTGATTGAATATTC | reverse primer for Δ(*pta*-*ackA*) construct (5'flank) |
| pta-ackA3 | CAATCAAGCAGAGAAAAGAA GTATGACTACCTGTTATTCGTC | forward primer for Δ(*pta*-*ackA*) construct (3'flank) |
| pta-ackA4 | ATATCAAGCTTATCGATACCGTCGACGCAGCAACCGCTG | reverse primer for Δ(*pta*-*ackA*) construct (3'flank) |
| ackA1-1 | TCAGGTTACCCGCATGCAAGATCTA TCTAGA GCAGCAACCGCTG | forward primer for Δ*ackA*1 construct (5'flank) |
| ackA1-2 | TTGGCTAGCCAGTTTATGCA GTATGACTACCTGTTATTCGTCTG | reverse primer for Δ*ackA*1 construct (5'flank) |
| ackA1-3 | CGAATAACAGGTAGTCATAC TGCATAAACTGGCTAGCC | forward primer for Δ*ackA*1 construct (3'flank) |
| ackA1-4 | AGTGTATATCAAGCTTATCGATACC GTCGAC CACTTGGTTAGCGAATGG | reverse primer for Δ*ackA*1 construct (3'flank) |
| ackA2-1 | AGTTGAAGTGATGAGATCTATCTAGA CAGTTTCGATAAGTGAGCC | forward primer for Δ*ackA*2 construct (5'flank) |
| ackA2-2 | AAATTTATAATGGCGCTATT GAAATAGTTATCCTGTTGTGGAG | reverse primer for Δ*ackA*2 construct (5'flank) |
| ackA2-3 | CACAACAGGATAACTATTTC AATAGCGCCATTATAAATTTGTG | forward primer for Δ*ackA*2 construct (3'flank) |
| ackA2-4 | ATATCAAGCTTATCGATACCGTCGAC TTCTCGCTTTAACTGGAAC | reverse primer for Δ*ackA*2 construct (3'flank) |

Tab. S2: comparison of the carbon fraction recovered by balancing carbon atoms and degree of reduction

| **Strain** | **Carbon fraction recovered in total products** | |
| --- | --- | --- |
|  | **Carbon balance** | **Degree of reduction balance** |
| *V. natriegens* WT | 99% | 100% |
| *V. natriegens* Δ*lldh* Δ*dldh* Δ*pfl* | 86% | 94% |
| *V. natriegens* Δ*lldh* Δ*dldh* Δ*pfl* Δ*ald* | 68% | 76% |
| *V. natriegens* Δ*lldh* Δ*dldh* Δ*pfl* Δ*ald* Δ*dns*::*pyc*_Cg_ | 88% | 100% |
